# The histone chaperone NAP1L3 is required for haematopoietic stem cell maintenance and differentiation

**DOI:** 10.1038/s41598-018-29518-z

**Published:** 2018-07-25

**Authors:** Yaser Heshmati, Shabnam Kharazi, Gözde Türköz, David Chang, Esmat Kamali Dolatabadi, Johan Boström, Aleksandra Krstic, Theodora Boukoura, Emma Wagner, Nadir Kadri, Robert Månsson, Mikael Altun, Hong Qian, Julian Walfridsson

**Affiliations:** 1 Center for Haematology and Regenerative Medicine, Department of Medicine, Karolinska Institutet, Karolinska University Hospital, Stockholm, Sweden; 2 Research Division of Translational Medicine and Chemical Biology, Department of Medical Biochemistry and Biophysics, Karolinska Institutet, Stockholm, Sweden; 3 Center for Haematology and Regenerative Medicine, Department of Laboratory Medicine, Karolinska Institutet, Karolinska University hospital, Stockholm, Sweden; 4 Hematology Center, Karolinska University Hospital, Stockholm, Sweden

## Abstract

Nucleosome assembly proteins (NAPs) are histone chaperones with an important role in chromatin structure and epigenetic regulation of gene expression. We find that high gene expression levels of mouse *Nap1l3* are restricted to haematopoietic stem cells (HSCs) in mice. Importantly, with shRNA or CRISPR-Cas9 mediated loss of function of mouse *Nap1l3* and with overexpression of the gene, the number of colony-forming cells and myeloid progenitor cells *in vitro* are reduced. This manifests as a striking decrease in the number of HSCs, which reduces their reconstituting activities *in vivo*. Downregulation of human *NAP1L3* in umbilical cord blood (UCB) HSCs impairs the maintenance and proliferation of HSCs both *in vitro* and *in vivo*. *NAP1L3* downregulation in UCB HSCs causes an arrest in the G0 phase of cell cycle progression and induces gene expression signatures that significantly correlate with downregulation of gene sets involved in cell cycle regulation, including E2F and MYC target genes. Moreover, we demonstrate that *HOXA3* and *HOXA5* genes are markedly upregulated when *NAP1L3* is suppressed in UCB HSCs. Taken together, our findings establish an important role for NAP1L3 in HSC homeostasis and haematopoietic differentiation.

## Introduction

Haematopoietic stem cells (HSCs) are rare multipotent blood-forming cells in the bone marrow giving rise to all lineages of mature cells throughout the postnatal life. The balanced self-renewal and differentiation capacity of HSCs is critical for preserving a stable source of HSCs while constantly replenishing all types of mature blood cells^1^. However, the mechanisms that orchestrate the balance remain poorly understood. It is well established that activation or suppression of lineage specific genes is tightly controlled by transcription factors that act in concert with epigenetic enzymes to determine the fates of HSCs^2^. These epigenetic enzymes catalyse the removal or addition of epigenetic modifications (e.g. DNA methylation and post-translational modifications of histone and histone variants) and alteration of the chromatin structure, without affecting the DNA coding sequence. Regulation of chromatin structure and inheritance of epigenetic information are instrumental in determining transcriptionally permissive or silenced chromatin states during the development and differentiation^2^.

The nucleosome assembly proteins (NAP) represent a family of evolutionarily conserved histone chaperones consisting of five members in mammals, having first been identified in mammalian cells^3^. These histone chaperones are thought to facilitate the import of H2A–H2B histone dimers from the cytoplasm to the nucleus^4,5^ and to regulate chromatin dynamics by catalysing the assembly or disassembly of nucleosomes^4,6–9^. More recently these histone chaperones have been implicated in the regulation of covalent histone modifications^10–14^ and exchange of histone variants in chromatin^15–19^. The composition and architecture of chromatin is important in all biological processes involving DNA^20^ and consequently the Nap1 family of proteins is important for a broad range of biological processes; including transcriptional regulation^10,14,21–34^, cell proliferation^35^, epigenetic transcriptional regulation^10,12,14,26,29,34,36,37^, DNA recombination^38–40^, chromosome segregation^18,41–43^ and DNA repair^42,44,45^. Moreover, the Nap1 family of histone chaperones has been associated with a role in the development of various organisms; including Arabidopsis^46,47^, C. elegans^48^, and Drosophila^49–51^, as well as in neural differentiation and function in mouse^52^.

However, the role of Nap1 proteins in haematopoiesis is largely unknown. Depletion of Nap1 in Xenopus embryos resulted in downregulation of alpha-globin and haematopoietic precursors genes, suggesting that Nap1 proteins have specific functions in haematopoiesis^53^. In this study, we investigate the *in vitro* and *in vivo* role of NAP1L3 in HSC activities and haematopoietic differentiation. Furthermore, we delineate the key transcriptional and signalling pathways underlying the role of NAP1L3 in haematopoiesis.

## Results

### *Nap1l3* is highly expressed in mouse haematopoietic stem cells

**Figure 1:**
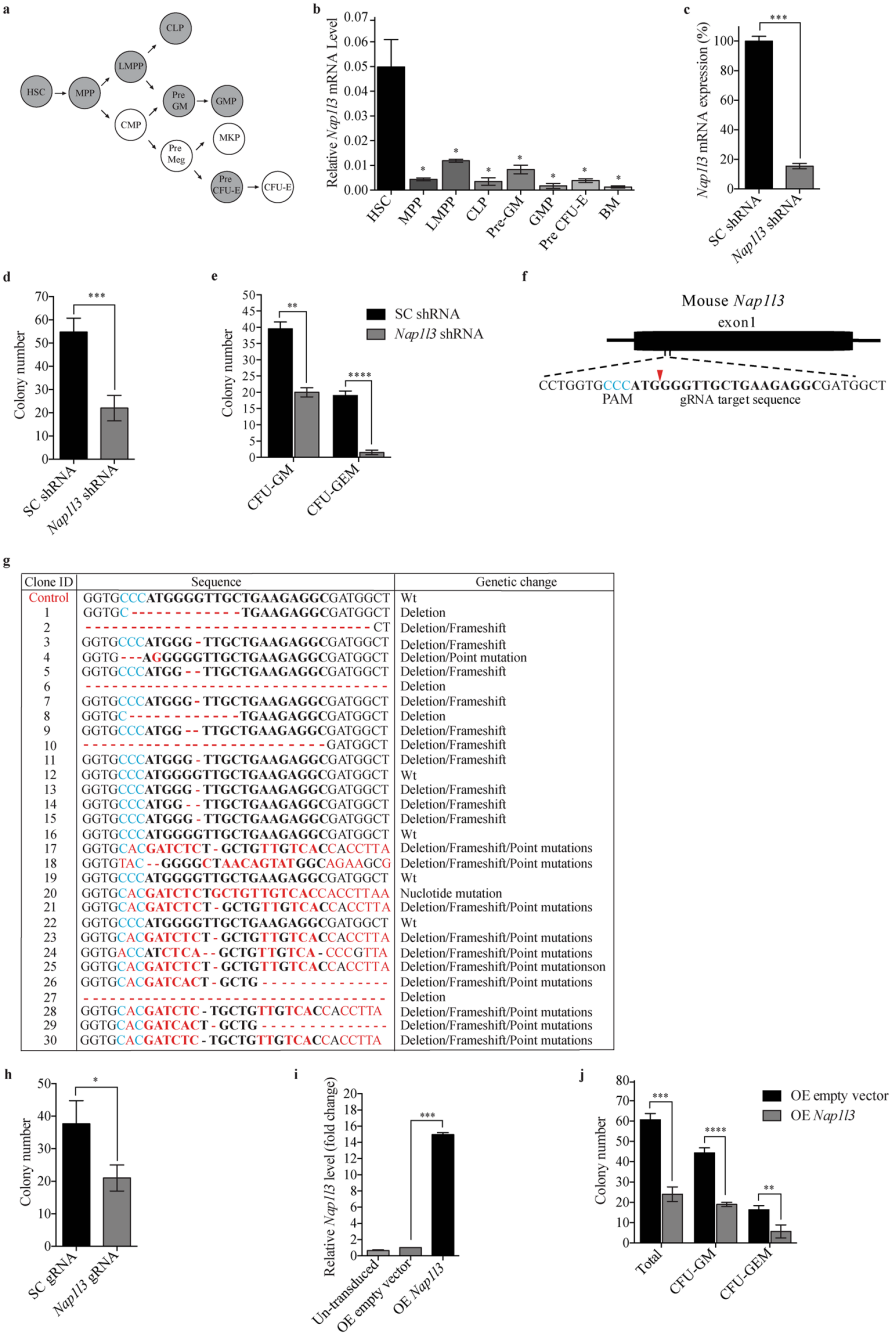
*Nap1l3* is predominantly expressed in murine haematopoietic stem cells and loss of function or overexpression impairs colony-forming capacity. (**a**) Illustration of 11 different primary murine HSPCs populations. The seven cell populations highlighted in grey were analysed in (**b**). (**b,c**) qPCR analysis showing *Nap1l3* mRNA levels (normalised to *Hprt*) of indicated cell populations (**b**) and of sorted LSK HSCs transduced with an shRNA against *Nap1l3* (*Nap1l3* shRNA), or a control vector (SC shRNA) (**c**). The data is represented as the mean ± s.e.m, *p < 0.05, ***p < 0.005 (unpaired t-test), n = 3. (**d,e**) The total colony numbers (**d**), and colony numbers of CFU-GM and CFU-GEM (**e**), formed from LSK HSCs transduced with *Nap1l3* shRNA (*Nap1l3* shRNA) or a control vector (SC shRNA) after ten days of clonal growth in methylcellulose. **p < 0.01, ***p < 0.005, ****p < 0.001 (unpaired t-test), n = 3. (**f**) Homology of the gRNA designed to target the murine *Nap1l3* gene (the protospacer adjacent motif [PAM] = blue letters, the Cas9 nuclease cutting site = red arrow and the gRNA target sequence = bold letters). (**g**) Sequencing results of 30 clones of the *Nap1l3* gene targeted by CRISPR-Cas9 in LSK HSCs (gRNA targeting sequence = bold letters, the PAM sequence = blue letters, and nucleotide changes relative to the WT *Nap1l3* = red dashes or letters), and the genetic changes are indicated in the last column (**h**) Colony numbers resulting from sorted LSK HSCs expressing Cas9 transduced with an inducible gRNA vector targeting *Nap1l3* (*Nap1l3* gRNA), or a control vector (SC gRNA). The colonies were analysed after 10 days of clonal growth in methylcellulose supplemented with doxycycline. The data is represented as the mean ± s.e.m., *p < 0.05 (unpaired t-test), n = 3. (**i**) qPCR analysis showing *Nap1l3* mRNA levels (normalised to *Hprt*) of enriched cKit^+^ HSCPs overexpressing *Nap1l3* (OE *Nap1l3*), an empty vector (OE empty vector) or un-transduced cells. The data is represented as the mean ± s.e.m., ***p < 0.005 (unpaired t-test), n = 3. (**j**) The total number of colonies, GM and GEM colonies formed of sorted cKit^+^ HSCPs overexpressing *Nap1l3* (OE *Nap1l3*) or an empty vector (OE empty), after ten days of clonal growth in methylcellulose. The data is represented as the mean ± s.e.m., **p < 0.01, ***p < 0.005, ****p < 0.001 (unpaired t-test).

*Nap1l3* has previously been shown to be expressed predominantly in haematopoietic stem cells (HSCs), compared to downstream haematopoietic progenies^54,55^, indicative of a potential functional role in primitive haematopoietic cells. To investigate the gene expression profile of *Nap1l3* in different populations of mouse haematopoietic stem and progenitor cells (HSPCs), we used a well-established flow cytometry protocol^56^ to determine *Nap1l3* mRNA levels in seven HSPCs cell populations from mouse bone marrow cells (BM); HSC (Lin^−^ Sca1^+^cKit^+^ [LSK^+^]CD105^+^CD150^+^), multi-potent progenitors (MPP; LSK^+^CD105^+^CD150^+^), lymphoid-primed multipotent progenitors (LMPP; LSK^+^Flk2^high+^), common lymphoid progenitors (CLP; Lin^−^IL7Ra^+^flk2^+^), *pre*-granulocyte-macrophage progenitors (pre-GM; LSK^−^CD41^−^CD150^−^CD105^−^), granulocyte-monocyte progenitors (GMP; LSK^−^CD41^−^CD150^−^FcgR^+^), and erythrocyte progenitors (pre-CFU E; LSK^−^CD41^−^CD105^+^) (Fig. 1a). Consistent with previous expression profiling experiments in mouse^55^ and human haematopoietic cells^54^, quantitative real time PCR (qPCR) showed that high *Nap1l3* mRNA expression was restricted to the HSC fraction, compared to the downstream haematopoietic progenitor cells and unfractionated BM cells (Fig. 1b).

### *Nap1l3* downregulation in primitive murine bone marrow cells reduces colony formation capacity

Given that high levels of *Nap1l3* gene expression were restricted to HSCs (Fig. 1b), we investigated the functional importance of *Nap1l3* in HSPCs differentiation and proliferation of primitive murine BM cells by shRNA-mediated loss of function studies. In this approach, the shRNA knockdown vectors were introduced by lentiviral transfer (i.e. transduction) to Lin^−^Sca^+^cKit^+^ (LSK) HSPCs sorted from lineage depleted BM cells. shRNA-mediated downregulation of *Nap1l3* resulted in a significant reduction (~85%) in mRNA levels compared to the negative scramble control vector expressing scrambled shRNA (Fig. 1c). Subsequently, downregulation of *Nap1l3* caused a marked reduction (approximately three-fold) in the total number of colony-forming units (CFUs) in the HSPCs compared to the control cells transduced with a negative control vector (Fig. 1d). Remarkably, downregulation of *Nap1l3* in LSK HSPCs led to a significant reduction of mixed myelo-erythroid CFUs (CFU-GEM) (ten-fold reduction) and a significant reduction in the number of granulocyte/macrophage CFUs (CFU-GM) (50% reduction), compared to cells transduced with a negative control vector (Fig. 1e). Consistent with this, downregulation of *Nap1l3* in LSK HSPCs with an independent shRNA, caused a similar reduction of total number of CFU-GEM and CFU-GM colony-forming units (Supplementary Fig. 1). This suggests that *Nap1l3* suppression preferentially affect the proliferation and survival of the more primitive HSPCs, which is in line with its restricted expression in HSCs.

### CRISPR-Cas9 mediated disruption of *Nap1l3* confirms a specific role in HSCs and differentiation

shRNA-mediated RNA interference (RNAi) can be associated with off-target effects if the shRNA itself possesses partial complementary to an undesired target elsewhere in the genome. Although the shRNAs targeting *Nap1l3* used in our present studies had no significant homology to any other open reading frame, we took advantage of highly specific CRISPR-Cas9 technology to ensure that the previously observed role of this nucleosome assembly enzyme in haematopoietic cells was specific. To test this, LSK HSPC BM cells from transgenic mice overexpressing Cas9 nuclease were isolated and transduced with inducible guide RNA (gRNA) vectors targeting the *Nap1l3* gene (Fig. 1f). Sanger sequencing of CRISPR-Cas9 targeted genomic DNA from the LSK HSPCs demonstrated that 25 out of in total 30 sequenced clones displayed genetic alterations in the *Nap1l3* gene. 17% of the cells carried significant deletions or continuous stretches of changed nucleotides (>10 base pair), and 60% of the cells displayed frameshift mutations, which likely will disrupt the gene function of *Nap1l3* (Fig. 1g). Consistent with our results from shRNA-mediated knockdown, CRISPR-Cas9 targeted disruption of the *Nap1l3* gene caused a significant reduction in number of total CFUs (>50% reduction) compared to the control cells after ten days of clonal growth (Fig. 1h), thus confirming a specific function of *Nap1l3* in colony forming capacity and proliferation of HSPCs.

### Overexpression of *Nap1l3* in primitive murine bone marrow cells reduces colony formation capacity

To further investigate the functional importance of *Nap1l3* in haematopoiesis, we used a lentiviral vector for stable exogenous expression of *Nap1l3* in cKit^+^ HSPCs. Constitutive overexpression of *Nap1l3* resulted in an approximately 14-fold increase in mRNA levels compared to endogenous levels in un-transduced cKit^+^ HSPCs and cKit^+^ cells transduced with an empty control vector (Fig. 1i). Surprisingly, as in our shRNA and CRISPR-Cas9 experiments (Fig. 1d,e and h), overexpression of *Nap1l3* in cKit^+^ HSPCs resulted in a significant reduction in the total number of CFUs, CFU-GM and CFU-GEM colonies, compared to control cells transduced with an empty vector after ten days of clonal growth and differentiation in methylcellulose (Fig. 1j).

### *Nap1l3* downregulation reduces the engraftment and frequency of primitive mouse cells *in vivo*

**Figure 2:**
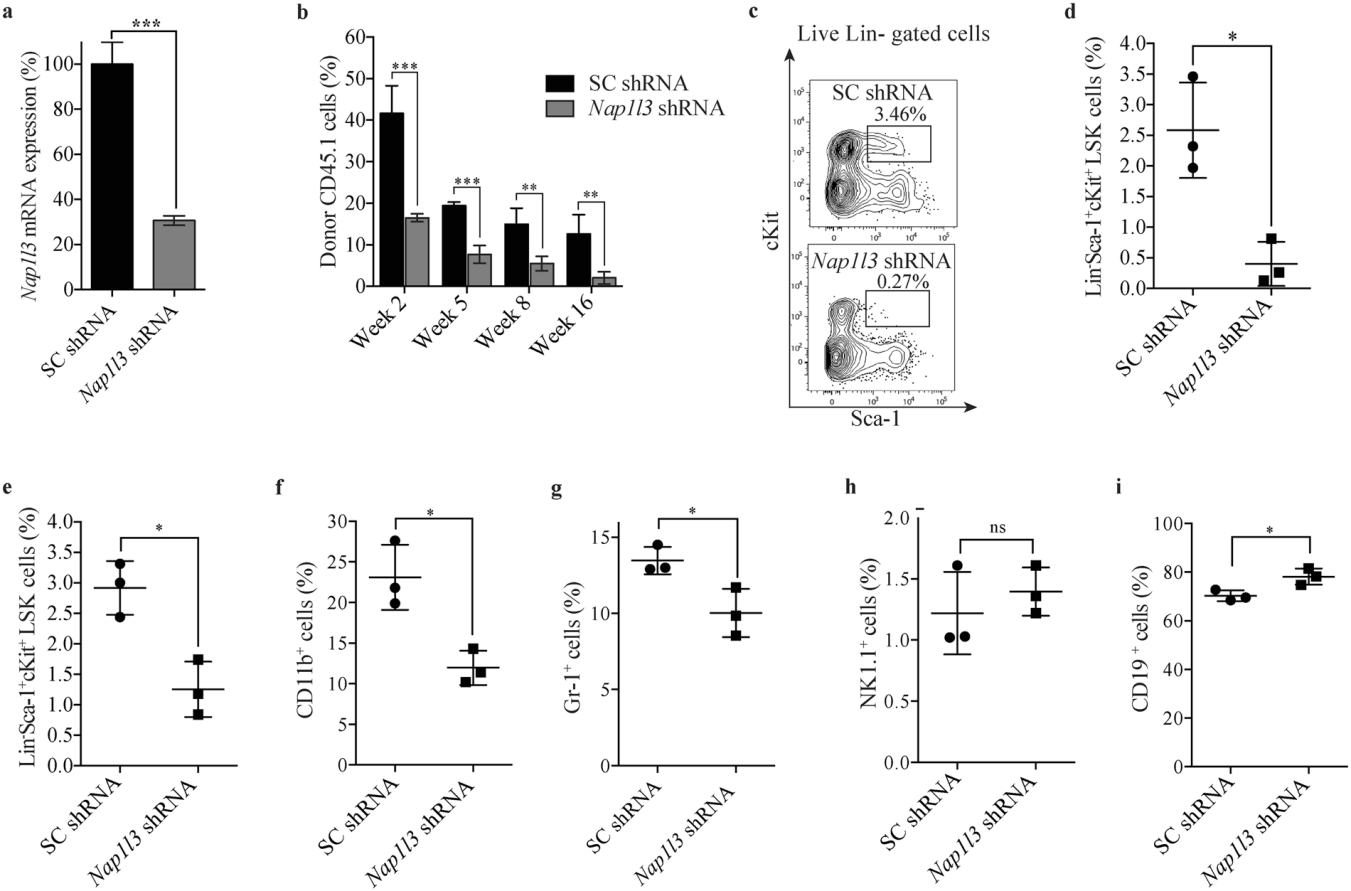
*Nap1l3* downregulation impairs maintenance of murine HSCs and blood lineage regeneration. (**a**) qPCR analysis of *Nap1l3* mRNA levels (normalised to *Hprt*) of sorted LSK HSCs transduced with an shRNA against *Nap1l3* (*Nap1l3* shRNA), or a control vector (SC shRNA). The data is represented as the mean ± s.e.m, *p < 0.05, ***p < 0.005 (unpaired t-test), n = 3. (**b**) Flow cytometric analysis of the percentage of CD45.1^+^ donor cells resulting from transduction of sorted cKit^+^ HSPCs transduced with *Nap1l3* shRNA (*Nap1l3* shRNA) or a control vector (SC shRNA) in peripheral blood of lethally radiated recipient mice, at two, five, eight and 16 weeks post transplantation. The data is represented as the mean ± s.e.m., **p < 0.01, ***p < 0.005 (unpaired t-test), n = 3. (**c**) Representative flow cytometric chart showing percentage of LSK HSC BM CD45^+^ donor cells, transduced with a negative control vector (SC shRNA, upper panel), or an shRNA vector targeting *Nap1l3* (*Nap1l3* shRNA, lower panel) in recipient mice five weeks post transplantation. The percentage of LSK HSCs is highlighted in each flow cytometry chart. (**d**,**e**) Percentage of donor derived LSK HSCs transduced with shRNAs against *Nap1l3* or a control vector. The cells were isolated from the bone marrow of recipient mice 5 weeks (**c**) and 16 weeks (**d**) after transplantation and analysed by flow cytometry. **p < 0.01 (unpaired t-test), n = 3. (**f**–**i**) Percentage of mature donor cells; CD11b^+^ myeloid (**f**), Gr-1^+^ granulocytes (**g**), NK1.1^+^ NK cells (**h**) and CD19^+^ B cells **(i**) after transduction of sorted cKit^+^ HSPCs transduced with shRNAs against *Nap1l3* (*Nap1l3* shRNA) or a control vector (SC shRNA). Bone marrow cells were isolated and analysed by flow cytometry 16 weeks post transplantation. ns = non-significant, *p < 0.05, (unpaired t-test).

To understand the importance of *Nap1l3* in haematopoiesis *in vivo*, we used a congenic murine bone marrow transplantation model. Accordingly, we transplanted sorted CD45.1^+^ cKit^+^ donor HSPCs transduced with either an shRNA vectors efficiently downregulating *Nap1l3* mRNA levels or a negative control vector expressing scrambled shRNA into lethally-radiated isogenic CD45.2^+^ wild-type (wt) C57BL/6 recipient mice (Fig. 2a). Longitudinal flow cytometric analysis of peripheral blood from the transplanted mice showed significant engraftment of donor cells transduced with negative control vectors at two, five, eight and 16 weeks post transplantation (15–45% of engraftment) (Fig. 2b). Conversely, transplanted cKit^+^ donor HSPCs transduced with an shRNA vector against *Nap1l3* resulted in a significant reduction (2–18% of engraftment) of engrafted cells compared to the control mice at two, five, eight and 16 weeks post transplantation (Fig. 2b).

Wanting to focus on studying the importance of *Nap1l3* in HSCs short-term maintenance *in vivo*, we used the same procedure as in Fig. 2b, and transplanted LSK HSCs with and without shRNA-mediated *Nap1l3* knockdown into lethally radiated recipient mice. Flow cytometric analysis of bone marrow cells from the recipient mice five weeks after transplantation revealed that downregulation of *Nap1l3* caused a distinct reduction of donor LSK cells (median percent of 0.4%), compared to transplanted LSK HSCs transduced with the negative control vector expressing scrambled shRNA (median percent of 2.6%) (Fig. 2c,d). To investigate the long-term effects of *Nap1l3* downregulation on LSK HSCs, we repeated our experiments and performed flow cytometric analysis at eight and 16 weeks post-translation. Consistent with the short terms results in Fig. 2d, downregulation of *Nap1l3* caused a significant reduction in donor LSK HSCs both at eight weeks (median percent of 0.2% in knockdown cells vs 1.8% in the control cells) and at 16 weeks (median percent of 1.2% in knockdown cells vs 3.0% in control cells), (Fig. 2e and Supplementary Fig. 2a). Altogether, this data suggests that *Nap1l3* plays an important role both in HSC short-term and long-term maintenance *in vivo*.

### *Nap1l3* downregulation in mouse HSPCs affects their capacity in blood lineage regeneration after transplantation

To investigate if the downregulation of *Nap1l3* affects the reconstitution of haematopoietic cells *in vivo*, we transplanted cKit^+^ donor HSPCs transduced with either *Nap1l3* shRNA or a negative control vector into lethally irradiated recipient mice, as in Fig. 2b–e. Flow cytometric analysis of mature blood cells showed a significant decrease in the frequency of myeloid cells (CD11b^+^), granulocytes (Gr-1^+^) (Fig. 2f,g), whereas the percentage of NK cells (NK1.1^+^) was not significantly changed (Fig. 2h), 16 weeks after transplantation. In contrast, the percentage of B cells (CD19^+^) was significantly increased in *Nap1l3* knockdown cells compared to the control cells (Fig. 2i). Apart from the results of the NK cells, comparable results were obtained after eight weeks after transplantation, as the results at 16 weeks after transplantation (Supplementary Fig. 2b–e). These data suggest that *Nap1l3* is required for HSC maintenance and haematopoietic restoration.

### *NAP1L3* is important for maintenance of human HSCs and proliferation *in vitro*

**Figure 3:**
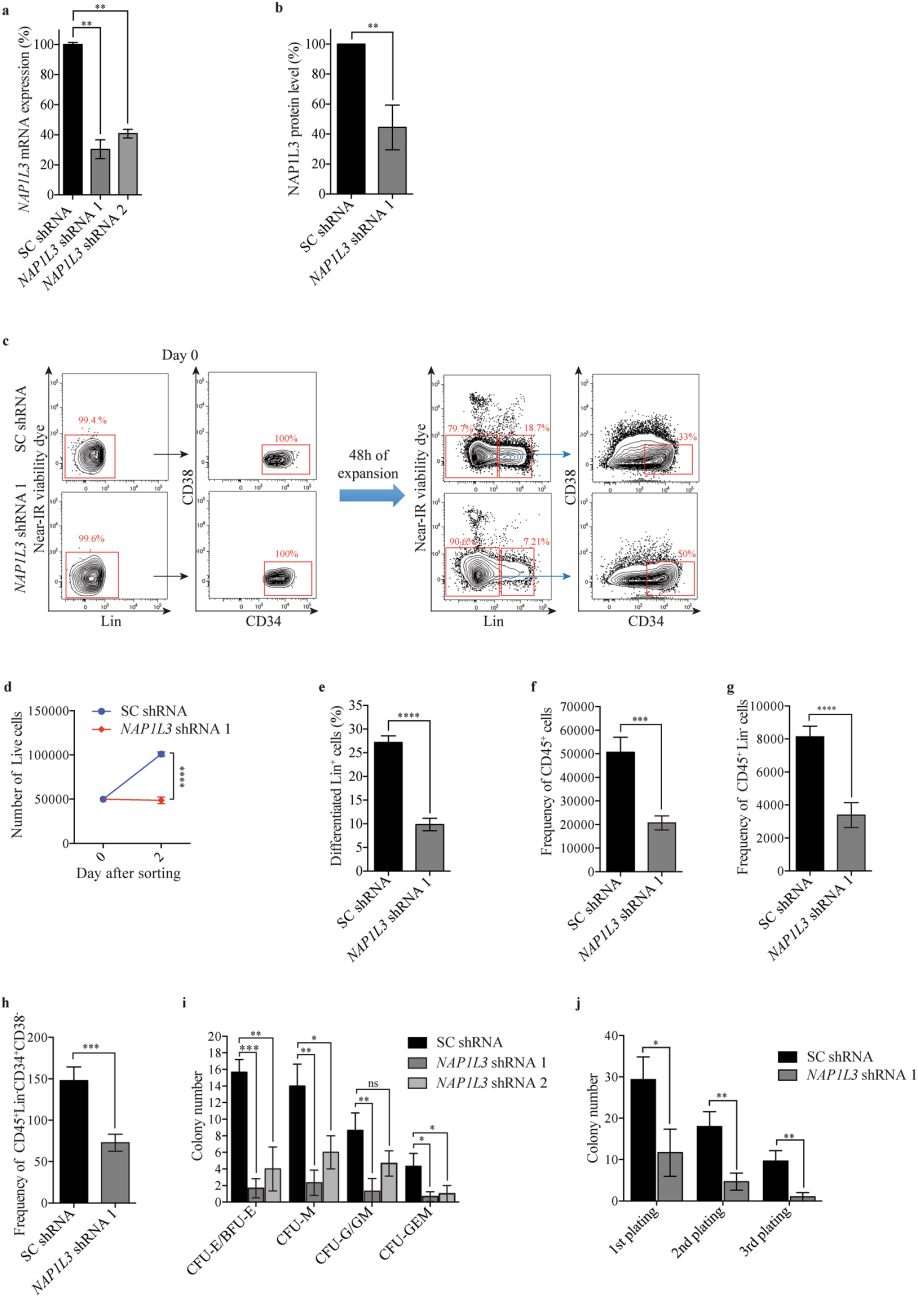
*NAP1L3* is important for proliferation and survival of human haematopoietic stem cells *in vitro*. (**a,b**) qPCR analysis showing *NAP1L3* mRNA levels (normalised to *UBC*) (**a**), or flow cytometric quantification of intracellular NAP1L3 protein levels (**b**), in of sorted (Lin^−^CD34^+^CD38^−^) UCB HSCs transduced with two independent shRNA vectors targeting *NAP1L3* (*NAP1L3* shRNA1 and 2) or a control vector (SC shRNA). The data is represented as the mean ± s.e.m., **p < 0.01 (unpaired t-test), n = 3. (**c**) Representative flow cytometry charts showing expression levels of lineage markers (Lin), CD34 and CD38 resulting from sorted (Lin^−^CD34^+^CD38^−^) UCB HSCs transduced with *NAP1L3* shRNA (*NAP1L3* shRNA), or a control vector (SC shRNA), immediately after sorting (Day 0, left panels) and 48 hours post sorting (right panels). The percentages of live Lin^−^, Lin^+^, and Lin^−^CD34^+^CD38^−^ cells are highlighted in red. (**d**) Absolute numbers of sorted (Lin^−^CD34^+^CD38^−^) UCB HSCs transduced with *NAP1L3* shRNA (*NAP1L3* shRNA), or a control vector (SC shRNA), immediately after transduction and sorting of 50,000 cells (day 0) and 48 hours after antibiotic selection (Day 2). The numbers of (Lin^−^CD34^+^CD38^−^) UCB HSCs was determined by flow cytometry according to (**c**). The data is represented as the mean ± s.e.m., ****p < 0.001 (unpaired t-test), n = 3. (**e**) Percentage of Lin^+^ UCBs transduced with *NAP1L3* shRNA (*NAP1L3* shRNA), or a negative control vector expressing scrambled shRNA (SC shRNA), determined by flow cytometry analysis according to (**b**), 48 hours post transduction and sorting of the cells. The data is represented as the mean ± s.e.m., ****p < 0.001 (unpaired t-test), n = 3. (**f–h**) Frequency of CD45^+^ (**e**), Lin^−^CD45^+^ (**f**) and Lin^−^CD34^+^CD38^−^ (**g**), after maintaining CD34^+^ enriched UCB HSPCs transduced with an shRNA against *NAP1L3* (*NAP1L3* shRNA) or a control vector (SC shRNA), for three weeks on murine LS/LS and M2-10B4 bone marrow stromal cells. The data is represented as the mean ± s.e.m., ***p < 0.005, ****p < 0.001 (unpaired t-test), n = 3. (**i**) Average number of CFU-E/BFU-E, CFU-M, CFU-G/GM, CFU-GEM, resulting from CD34^+^ enriched UCBs transduced with two individual shRNA vectors targeting *NAP1L3* (*NAP1L3* shRNA) or a control vector (SC shRNA), after 14 days of clonal growth in methylcellulose. The data is represented as the mean ± s.e.m., ns = non-significant, *p < 0.05, **p < 0.01, ***p < 0.005 (unpaired t-test), n = 3. (**j**) Serial replating assay of colony formation by control or *NAP1L3* transduced HSPC UCBs. CFUs were scored every 14 days and the cells were replated in triplicate. The data is represented as the mean ± s.e.m., *p < 0.05, **p < 0.01 (unpaired t-test), n = 3.

We next investigated if NAP1L3 was also important for human HSC maintenance. Thus, to investigate early cellular responses of primitive UCBs to downregulation of *NAP1L3*, we transduced sorted human (Lin^−^CD34^+^CD38^−^) UCB enriched HSCs (hereinafter referred to as UCB HSCs) with either two independent shRNAs against *NAP1L3* or a negative control vector. qPCR analysis of mRNA levels (Fig. 3a) and flow cytometry analysis of intracellular protein levels (Fig. 3b and Supplementary Fig. 3), demonstrated an efficient downregulation of NAP1L3 gene expression levels. Flow cytometric analysis revealed that UCB HSCs transduced with a control vector underwent a cell doubling after 48 hours of culture in suspension, whilst *NAP1L3* downregulated-HSCs displayed impaired cell proliferation (Fig. 3c,d), suggesting that *NAP1L3* is required for the *in vitro* proliferation of UCB HSCs. In addition, we also observed a reduction of the frequency of cells expressing mature cell lineage markers (Lin^+^) derived from the *NAP1L3* shRNA targeted UCBs as compared to the control cells (Fig. 3e).

To investigate the long-term effects of *NAP1L3* suppression in UCB HSCs *in vitro*, we transduced CD34^+^ enriched HSPC UCBs with vectors targeting *NAP1L3* or control vectors and co-cultured these cells with murine SL/SL and M2-10B4 bone marrow stromal cell lines, allowing for the maintenance and functional detection of primitive human haematopoietic cells^57^. Consistent with the results in murine haematopoietic cells (Fig. 1d,e,h and j) and in the short-term experiment using human UCBs (Fig. 3b–d), flow cytometric analysis revealed a significant reduction in CD45^+^ (Fig. 3f), Lin^−^CD45^+^ (Fig. 3g), and UCB HSCs (Fig. 3h), after maintaining the cells for three weeks on the stromal cells.

Wanting to further investigate the role of NAP1L3 in proliferation and differentiation of haematopoietic cells in human cells, we transduced CD34^+^ enriched UCB HSPCs with one of two shRNAs against human *NAP1L3* or a negative control vector. After 14 days of clonal growth in methylcellulose, a significant reduction in the numbers of; burst-forming unit erythroid cells (CFU-E/BFU-E, 90%), macrophages (CFU-M, 50%), granulocytes/macrophages (CFU-G/GM, 50%), and mixed myelo-erythroid cells (CFU-GEM, 75%) were observed for both shRNAs compared to the control cells transduced with a negative control vector, with the exception of *NAP1L3* shRNA 2 in CFU-G/GM cells, which did not yield a significant reduction (Fig. 3i). Strikingly, serial replating of HSPCs expressing shRNAs against *NAP1L3* resulted in a significant reduction in the total number of colonies in the first plating, which was further reinforced in the second and third replating of the cells, compared to control cells (Fig. 3j).

These data indicate that the importance of mouse *Nap1l3* in maintenance of HSCs is conserved in human UCBs.

### *NAP1L3* downregulation induces an arrest in the G0 phase of the cell cycle and apoptosis in UCBs

**Figure 4:**
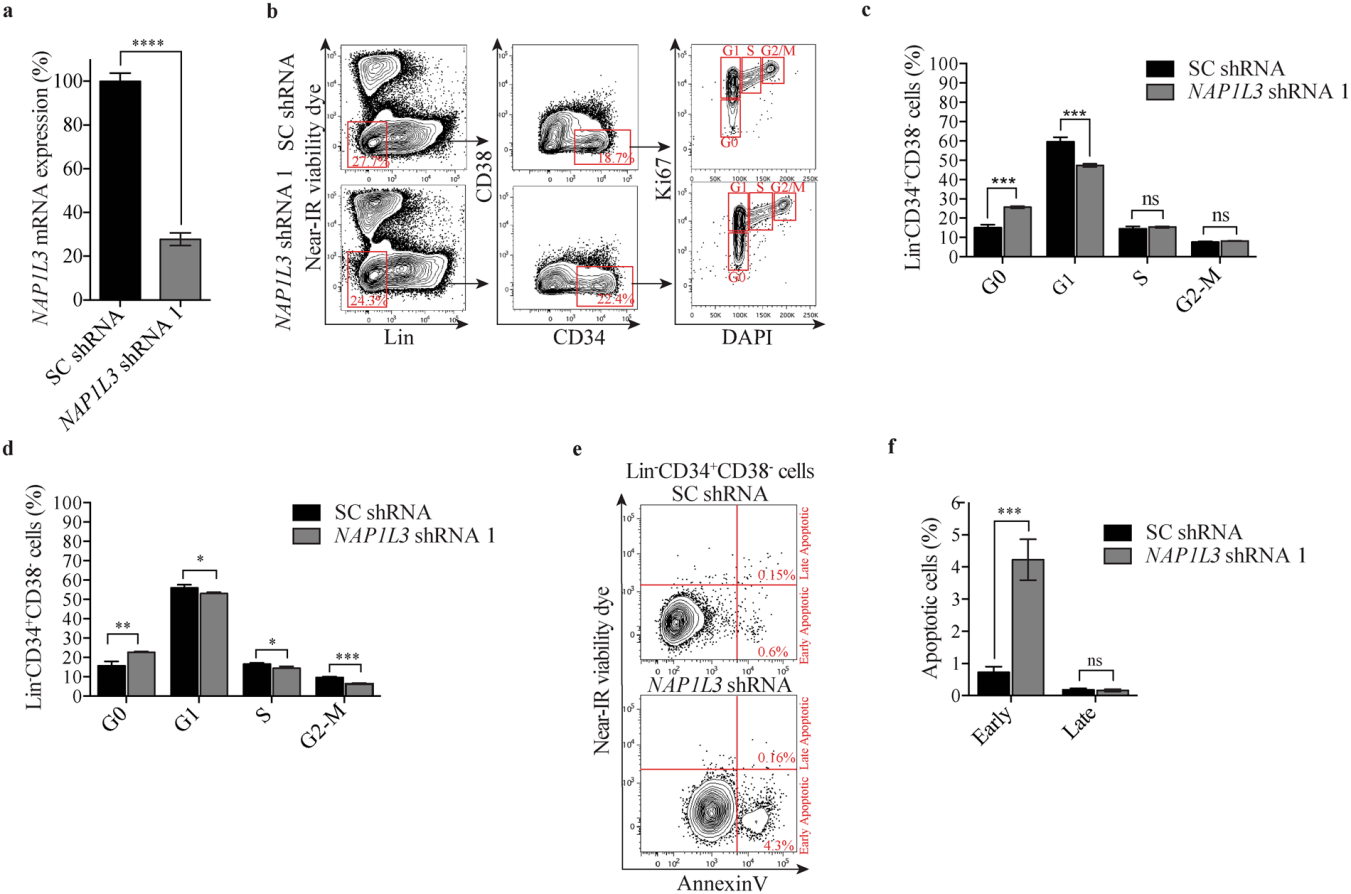
*NAP1L3* downregulation causes an arrest in the G0 phase of the cell cycle and induces apoptosis. (**a**) qPCR analysis showing *NAP1L3* mRNA levels (normalised to *UBC*) in sorted (Lin^−^CD34^+^CD38^−^) UCB HSCs transduced with shRNA vectors targeting *NAP1L3* (*NAP1L3* shRNA) or control vectors (SC shRNA). The data is represented as the mean ± s.e.m., **p < 0.01 (unpaired t-test), n = 3. (**b**) Representative flow cytometry charts of intracellular Ki67 and DNA content (DAPI) staining in (Lin^−^CD34^+^CD38^−^) UCB HSCs transduced with *NAP1L3* shRNA (*NAP1L3* shRNA), or a control vector (SC shRNA), 5 days post transduction. The percentages of Lin^−^ and CD34^+^CD38^−^ UCBs and the different phases of the cell cycle are highlighted in red. (**c**,**d**) The proportion of (Lin^−^CD34^+^CD38^−^) UCB HSCs transduced with *NAP1L3* shRNA (*NAP1L3* shRNA) or a control vector (SC shRNA), in the different phases of the cell cycle four days (**c**) and six days (**d**), post transduction. The data is presented as mean ± s.e.m., ns = non significant, *p < 0.05, **p < 0.01, ***p < 0.005 (unpaired t-test), n = 3. (**e**) Representative flow cytometry charts of (Lin^−^ CD34^+^CD38^−^) HSC UCBs transduced with *NAP1L3* shRNA (*NAP1L3* shRNA), or a control vector (SC shRNA), stained with near-IR viability control and Annexin V, to detect apoptotic cells, five days post transduction. The percentage of cells in each population is highlighted in red. (**f**) The proportion of early or late apoptotic (Lin^−^CD34^+^CD38^−^) UCB HSCs transduced with *NAP1L3* shRNA or a control vector, two days after sorting live cells. The data is presented as mean ± s.e.m., ns = non significant, ***p < 0.005, (unpaired t-test), n = 3.

To investigate the cellular mechanisms by which NAP1L3 is important for haematopoiesis, we sorted UCB HSCs and transduced the cells with shRNAs against *NAP1L3* or negative control vectors (Fig. 4a). *NAP1L3* knockdown resulted in a marked accumulation of UCB HSCs in the G0 phase of the cell cycle (25%) compared to control cells (15%), at four days after transduction. In addition, we observed a similar reduction of *NAP1L3* knockdown cells in G1 (48%) compared to that of the control cells (60%), whereas no significant change was observed for the S or G2/M phases (Fig. 4b,c). After six days of propagation, a similar proportion of the UCB HSCs resided in the G0 phase of the cell cycle as compared to four days after transduction, but this time we also observed a significant reduction of cells in the S and G2/M phases (Fig. 4d).

UCB HSCs transduced with a negative control vector displayed low levels of both early apoptotic cells (Annexin V^+^NIR^−^) (0.6%) and late apoptotic cells (Annexin V^+^NIR^+^) (0.2%) after maintaining the sorted live cells for two days in culture (Fig. 4e,f). In contrast, UCB HSCs transduced with shRNAs against *NAP1L3* resulted in a marked increase in the percentage of early apoptotic cells (4.5%), but no significant increase in late apoptotic cells (0.9%) (Fig. 4e,f).

Together, this data suggests that downregulation of *NAP1L3* causes a growth arrest of UCB HSCs in the G0 phase of cell cycle progression and triggers apoptosis, which might explain the observed reduction in HSC numbers and activities.

### *NAP1L3* downregulation is important for HSCs maintenance and SCID-repopulation capacity *in vivo*

**Figure 5:**
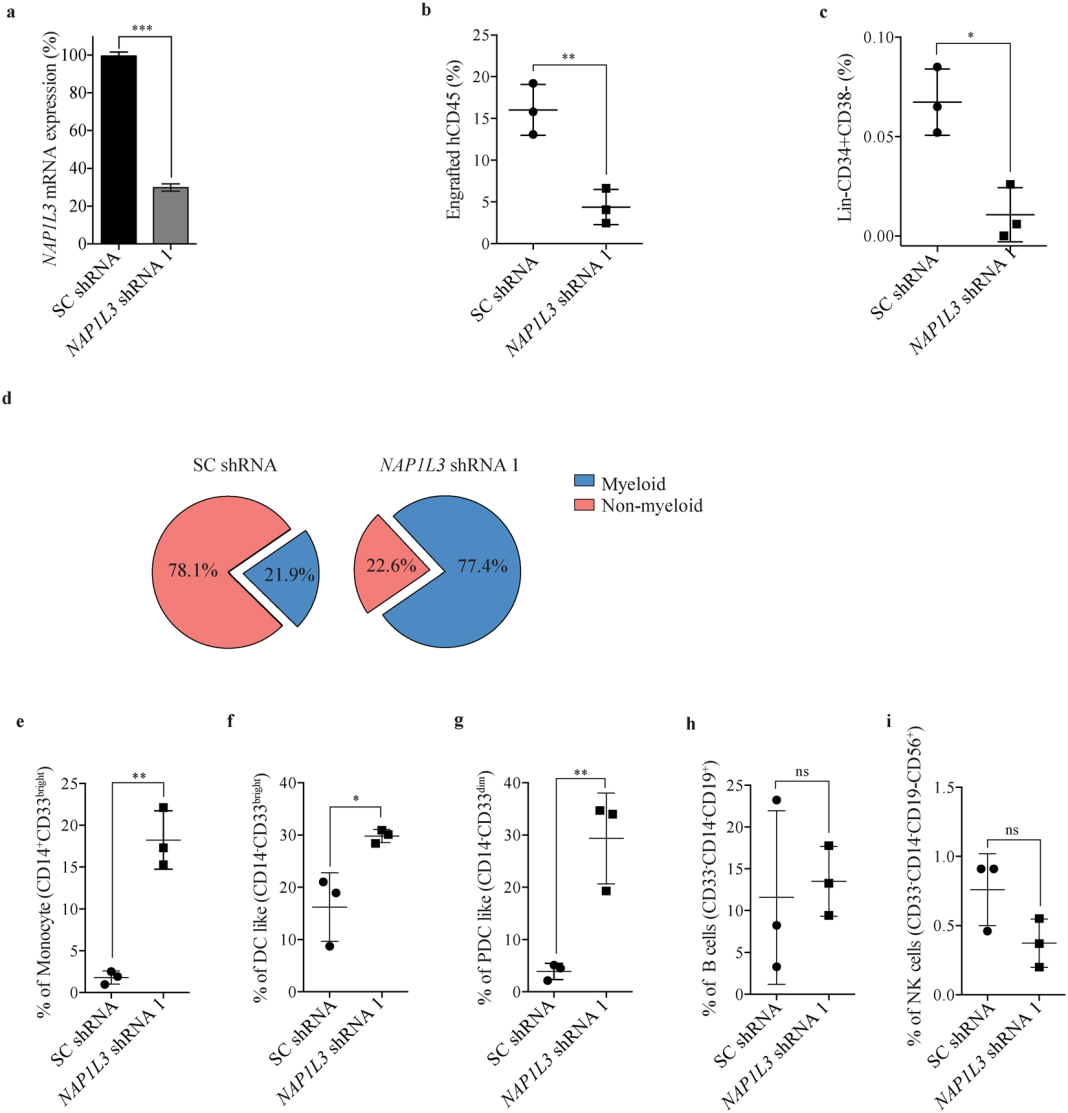
*NAP1L3* suppression reduces HSC maintenance and affects differentiation *in vivo*. (**a**) Bar chart showing relative mRNA levels of *NAP1L3* determined by qPCR of enriched CD34^+^ UCBs, transduced with shRNA vectors targeting *NAP1L3* (*NAP1L3* shRNA) or negative control vector (SC shRNA). *NAP1L3* mRNA expression levels were normalised to the *UBC* gene mRNA levels. The data is represented as the mean ± s.e.m., ***p < 0.005 (unpaired t-test), n = 2. (**b**,**c**) Percentage of engraftment of CD45^+^ (**b**) or Lin^−^CD34^−^CD38^+^ HSC UCBs (**c**), transduced with shRNAs against *NAP1L3* or a control vector. The UCBs were isolated from the bone marrow of recipient NSG-SGM3 mice at 16 weeks. The data is analysed by flow cytometry and is represented as the mean ± s.e.m.*p < 0.05, **p < 0.01 (unpaired t-test), n = 3. (**d**) Pie charts depicting the proportion of myeloid (monocytes, dendritic like cells, plasmacytoid dendritic cells) and non-myeloid BM cells (lymphoid B cells, and NK cells, from the same mice as in (**b**) and (**c**). (**e**–**i**) Percentage of engraftment of monocytes (**e**), dendritic like cells (**f**), plasmacytoid dendritic cells (**g**), B cells (**h**), and NK cells (**i**), transduced with shRNAs against *NAP1L3* or a control vector. The data is analysed by flow cytometry and is represented as the mean ± s.e.m. ns = non significant, *p < 0.05, **p < 0.01 (unpaired t-test), n = 3.

To investigate the importance of human NAP1L3 in haematopoiesis we transplanted CD34^+^ enriched HSPC UCBs transduced with shRNA vectors against *NAP1L3*, or a negative control vector expressing scrambled shRNAs (Fig. 5a), into a humanised NSG-SGM3 xenograft mouse model of haematopoiesis^58^. Flow cytometric analysis of UCB BM cells transduced with a negative control vector revealed a robust engraftment of human nucleated haematopoietic cell (CD45^+^) in transplanted recipient mice at 16 weeks after transplantation (Fig. 5b). In contrast, when analysing UCB BM CD45^+^ cells from recipient mice transplanted with shRNA vectors efficiently knocking down *NAP1L3* (Fig. 5a), a notable reduction of engraftment was observed using flow cytometric analysis (median 75% compared to control mice) (Fig. 5b). When analysing the UCB HSCs transduced with *NAP1L3* shRNAs, we observed a significant reduction of these primitive cells in the NSG-SGM3 recipient mice, compared to the control cells transduced with a negative control vector (Fig. 5c).

To determine the importance of *NAP1L3* in cell differentiation of UCB BM cells *in vivo* we further analysed the engraftment of myeloid (monocytes, dendritic like cells, plasmacytoid dendritic cells) and non-myeloid BM cells (lymphoid B-cells, NK cells), in the transplanted mice. Flow cytometric analysis of UCBs transduced with *NAP1L3* shRNA vectors revealed a dramatic increase in the proportion of myeloid cells, compared to UCBs transduced with non-targeting vectors (p-value = 0.0006), and a resulting reverse correlation of non-myeloid cells (Fig. 5d). In agreement with the increased proportion of myeloid UCBs when *NAP1L3* is downregulated, we observed a significant increase of monocytes (Fig. 5e), dendritic like cells (DC like) (Fig. 5f), and plasmacytoid dendritic cells (PDC) (Fig. 5g), compared to the control cells. Although the proportion of lymphoid cells was substantially different between *NAP1L3* knockdown cells and control cells (Fig. 5d), we did not observe any significant difference in engraftment when we compared the proportion of B cells (Figure H) and NK cells (Figure I).

Collectively, these data show that *NAP1L3* has an important role in engraftment and maintenance of human HSCs and cell differentiation *in vivo*.

### Downregulation of *NAP1L3* induces suppression of transcriptional programs correlating to cell cycle progression

The importance of *NAP1L3* in haematopoiesis is likely to be attributed to its function in regulating chromatin structure and consequently transcriptional gene regulation. To investigate the molecular mechanisms by which *NAP1L3* contributes to human HSC function and haematopoietic differentiation, we performed RNA sequencing (RNA-Seq) and global expression profiling analysis of sorted human UCB HSCs, transduced with shRNA vectors targeting *NAP1L3*, compared to cells transduced with a negative control vector, 72 hours post transduction of the cells.

**Figure 6:**
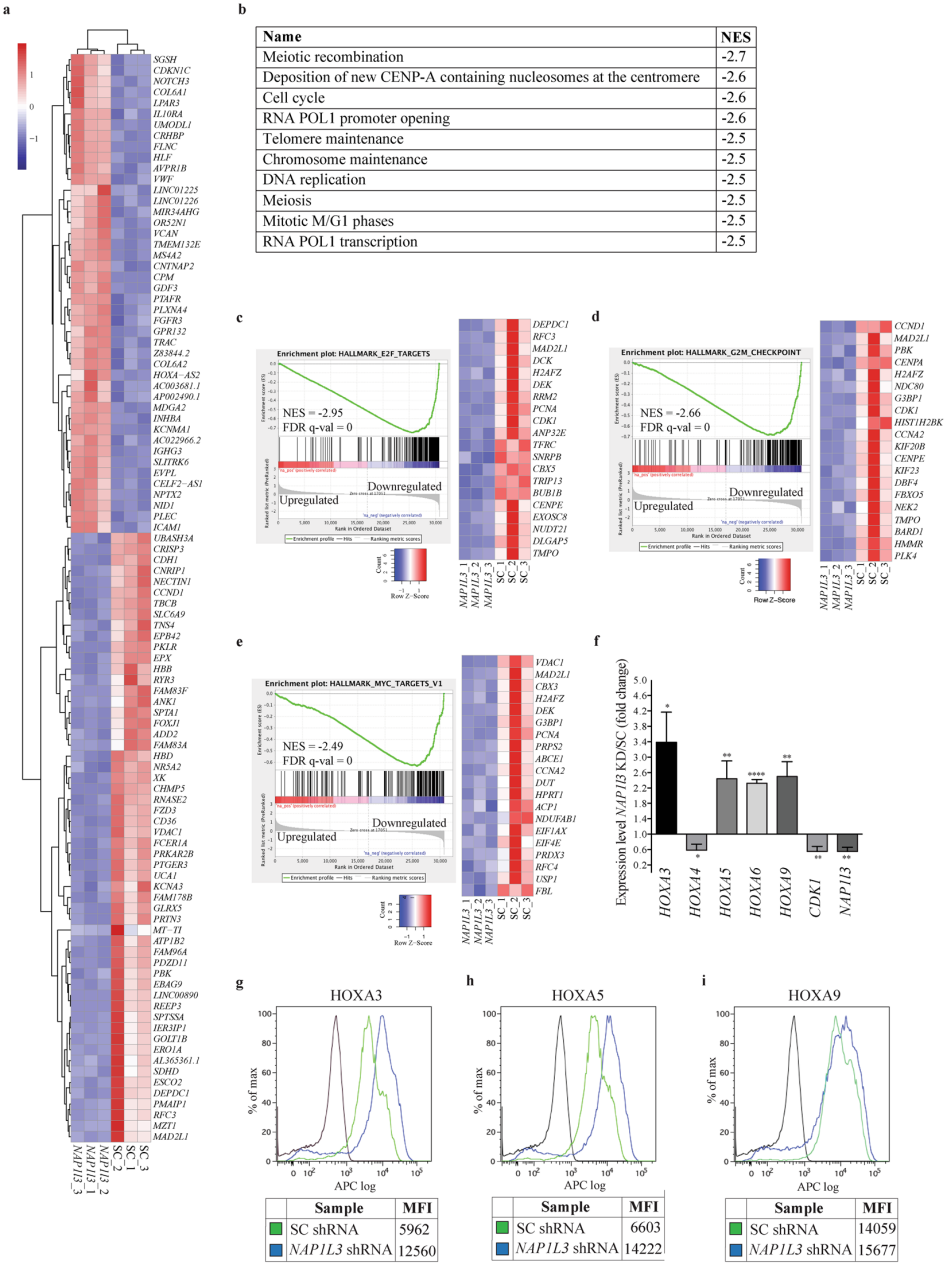
*NAP1L3* downregulation in cord blood cells induces an expression profile linked to cell cycle progression. (**a**) Heatmap of the top 100 genes differentially expressed in (Lin^−^CD34^+^CD38^−^) UCB HSCs transduced with shRNA vectors against *NAP1L3*, relative to a control vector, 72 hours post transduction. Up regulated genes are shown in red and down regulated genes are shown in blue. The data represents clustering of the individual experiments, n = 3. (**b**) List of the ten most significant correlations between the predefined MSigDB GO Biocarta gene set pathways and the gene expression changes resulting from shRNA-based downregulation of *NAP1L3*, relative to UCB HSCs transduced with negative control vectors. Normalised enrichment score (NES) for each pathway is shown in the right column. (**c**–**e**) Enrichment plots of gene set enrichment analysis (GSEA) of gene expression profiling changes in response to *NAP1L3* knockdown in UCBs, demonstrating significant negative correlations with E2F targets (**c**), G2/M checkpoint (**d**) and MYC targets (**e**). The bar charts represent the top ranked correlations in the predefined MSigDB: H hallmark collections. The heatmap on the right in Fig. 5c–e shows the relative level of gene expression (red = high, blue = low) of the leading edge gene subset. NES scores, FDR-q values and up or downregulated genes as a consequence of *NAP1L3* knockdown compared to control cells, are indicated in the diagrams (**f**). qPCR analysis of mRNA levels (normalised to *UBC*) of genes showing changes in expression in RNA-Seq of CD34^+^ HSPC UCBs cells transduced with shRNA against *NAP1L3*, relative to cells transduced with control vectors, 72 hours post transduction. The data is represented as the mean ± s.e.m., *p < 0.05, **p < 0.01, ****p < 0.001 (unpaired t-test), n = 3. (**g**–**i**) Flow cytometric quantification of mean fluorescence intensity (MFI) of intracellular HOXA3 (**g**), HOXA5 (**h**) and HOXA9 (**i**), protein levels, of CD34^+^ HSPC UCBs cells transduced with shRNA against *NAP1L3* or cells transduced with control vectors, 72 hours post transduction.

Analysis of the RNA-Seq data showed a high degree of correlation between the triplicates and a significant differentiation between the UCB HSCs transduced with shRNAs against *NAP1L3*, compared to their control counterpart cells (Fig. 6a). We identified 310 genes which mRNA levels were significantly upregulated and 244 genes that were downregulated in *NAP1L3* knockdown cells compared to control cells (1 or log2FC < −1; p > 0.05; p-value adjusted for multiple testing p < 0.05) (Supplementary Table S1).

To identify biological processes and pathways involving *NAP1L3*, which are important for the function of UCB HSCs, we performed gene set enrichment analysis (GSEA)^59^. GO-term analysis of Biocarta gene set pathways revealed that the top ten most significant correlations were all negatively correlated to cell cycle regulation, chromosome function, recombination and replication (Fig. 6b). Moreover, additional GSEA analysis, with a focus on molecular signatures, showed that the most significant correlations to the transcriptome profile, due to *NAP1L3* downregulation in UCB HSCs, encompassed a gene set enriched for; E2F targets (false discover rate (FDR) q value = 0.0, normalised enrichment score (NES) = −3.0) (Fig. 6c), a signature of genes involved in G2/M checkpoint (FDR q value = 0.0, NES −2.7) (Fig. 6d) and MYC targets (FDR q value = −0.0, NES −2.5) (Fig. 6e). In addition to the described pathways and gene signatures, we also observed that many of the genes in the *HOXA* cluster were significantly upregulated in the RNA-seq data (Supplementary Table S1).

To validate the RNA-Seq data and the GSEA analysis, we performed additional shRNA-mediated suppression of *NAP1L3* in UCB HSCs. qPCR analysis of mRNA expression revealed that nearly all the genes selected for validation (except for *HOXA4*) showed the same trend and comparable levels of changes to the RNA-seq results in human UCB HSCs transduced with shRNA vectors targeting *NAP1L3*, compared to cells transduced with a negative control vector (Fig. 6f). Further flow cytometric assessment of the *NAP1L3* shRNA-targeted UCBs with a significant reduction in NAP1L3 protein levels in Fig. 3b and Supplementary Fig. 3, demonstrated a more than two-fold upregulation of HOXA3 and HOXA5 of protein levels, whereas no significant change was observed for HOXA9 (Fig. 6g–i).

To investigate how enforced expression of *NAP1L3* affected HOXA3, HOXA5 and HOXA9 gene expression levels, qPCR and flow cytometric analysis was performed on UCBs transduced with lentiviral vectors expressing of NAP1L3. The analysis revealed a close to two-fold increased expression of NAP1L3 on both mRNA and protein levels over endogenous levels, but no significant change was observed for the HOXA genes (Supplementary Fig. 4c–e).

In conclusion, both the RNA-Seq analysis and the validation showed that downregulation of *NAP1L3* in UCB HSCs induces gene expression signatures associated to cell cycle progression and *HOXA* gene expression.

## Discussion

Regulation of chromatin dynamics is important in all biological processes where DNA accessibility needs to be regulated^60^. Histone chaperones constitute a family of chromatin regulators that have an important function in these processes. Our studies identified a novel role of the histone chaperone *NAP1L3* in early haematopoiesis. Moreover, we found *NAP1L3* to be highly expressed in HSCs, and showed that loss of function of *NAP1L3* in primitive human UCB HSCs had a remarkable impact on the survival and proliferation of HSCs and cell differentiation *in vivo*. These effects appears to be associated to its role in the transcriptional regulation of genes, which in turn encode the proteins required for proper cell cycle progression and differentiation.

The Nap1 family of histone chaperones has previously been implicated in cell cycle regulation via functional and physical interactions with mitotic cyclins in both budding yeast and frogs^35,61,62^. In addition, Nap1 null mutants in *S. pombe* cause a delay in mitosis^63^. In contrast, we here show that NAP1L3 is required for maintenance of HSCs and that its downregulation induces an arrest in the G0 phase of the cell cycle. Whilst further studies are required to delineate if the induced growth arrest due to *NAP1L3* downregulation is reversible (quiescence) or irreversible (senescence), it is possible that NAP1L3 controls HSC cell-cycle entry and proliferation in a normal setting. Nevertheless, this seems to, at least in part, be mediated by transcriptional regulation of signalling pathways with essential functions in cell cycle progression, including MYC and E2F targets. Consistent with this notion, the NAP1 family have previously been shown to physically interact with and to augment p300/CBP gene transcription of E2F1 and p53, both of which play a critical role in cell cycle progression and apoptosis^29^. The importance of NAP1L3 in primitive haematopoietic cells may represent its multifaceted functions in various signalling pathways (e.g. cell cycle progression, apoptosis via MYC and E2F targets). On the other hand, it may be reflective of a critical function in one signalling pathway causing secondary effects in several other processes. Further studies are clearly needed to fully understand the various aspects of *NAP1L3* function in regulating HSC fates. Nonetheless, our data suggest that NAP1L3 is involved in transcriptional regulation of HSC cycling.

Although high expression of *NAP1L3* is restricted to HSCs, its importance in haematopoiesis might also be related to HSC reconstitution activities since gene knockdown of the human *NAP1L3* cause abnormal mature blood lineage regeneration after transplantation. One explanation for this phenomenon might be that NAP1L3 establishes a chromatin landscape in HSCs, which is crucial for primitive haematopoietic cell function that is further propagated via epigenetic mechanisms to more mature haematopoietic cells. This is supported by the fact that the NAP1 family of proteins has been reported to be involved in development and cell differentiation in various species^46,49–52,64–66^, tissues, and cell types^47,67,68^, including haematopoiesis in Xenopus^53^ and epigenetic transcriptional regulation^10–14^. Together these findings show that the Nap1 family of proteins are emerging as regulators of development and cell differentiation. Surprisingly, our global transcriptome analysis in *NAP1L3* downregulated UCBs did not identify strong correlations to signalling pathways with a known role in haematopoietic differentiation. However, the mRNA levels of five out of 13 HOXA genes are significantly upregulated in UCBs upon suppression of *NAP1L3* and two out of three also show increased protein levels. Although the mechanistic basis for this remains to be determined, it is possible that the important role of NAP1L3 in HSCs maintenance and haematopoietic restoration shown in this study, may involve epigenetic transcriptional regulatory mechanisms of the HOXA genes in a similar way to what has been previously reported^14,26,29,34,36,37,42^. Nonetheless, it is known that the *HOXA* cluster of genes are highly expressed in uncommitted HSPCs compared to mature cells and they play a pivotal role in self-renewal and haematopoietic differentiation^69^. Although the mechanisms by which NAP1L3 contribute to regulation of the HOXA gene cluster in haematopoiesis is unknown, this correlation may provide a plausible explanation for the observed effects in differentiation.

Surprisingly, both overexpression and loss-of-function of *Nap1l3* produce similar phenotypes in primitive haematopoietic and differentiated cells. A likely explanation for this is that forced overexpression at non-physiological levels of the protein causes dominant negative effects. It is well established that over-enforced expression of wild-type proteins can cause a mutant phenotype due to competition with other macromolecules and/or non-functional subassemblies/disassembly’s, leading to a reduced function of the protein^70^. However, enforced expression of *NAP1L3*, which cause effects in haematopoietic stem cell function and maintenance, do not affect HOXA gene regulation, suggesting that deregulation of other downstream effectors may play a critical role in these processes. Regardless, the overexpression and subsequent phenotype in HSCs and cell differentiation confirms the importance of *Nap1l3* in haematopoiesis.

In contrast to many other transcription factors and epigenetic enzymes with a role in HSC function and cellular differentiation, *NAP1L3* has not been reported to be genetically mutated in haematological malignancies^71^ or to be associated with clinical outcomes^2,54,72^. However, *NAP1L3* is significantly overexpressed in AML HSPCs compared to normal HSPCs^54^. Although the transplanted animals in this study did not display any features of malignant transformation into leukaemia, this overexpression in AML cells together with a strong association to *HOXA* genes, cell cycle progression, *MYC* and *E2F*, indicate that NAP1L3 may have a role in leukemogenesis. Future studies will investigate whether *NAP1L3* contributes to cellular transformation or maintenance of AML by modulating cell cycle progression and *HOXA*, *MYC*, and *E2F* gene expression.

## Methods

### Isolation and culturing of primary cells

Sorted primary murine cKit^+^ and LSK cells were cultured in SFEMII media (Stemcell technology) supplemented with; rhFlt3/Flk-2 ligand (Stemcell technology), rhTPO (Stemcell technology), rhIL-6 (R&D system), rmIL-3 (R&D systems) and rmSCF (R&D systems) at a concentration of 20 ng/mL. UCB samples were provided by the Karolinska Hospital (Stockholm, Sweden) with informed consent from the parents and all investigation has been performed according ethical standards and to the declaration of Helsinki and to national and international guidelines. The experiments on the UCB samples were approved by The Regional Ethical Review Board in Stockholm (Dnr: 2012/480-31/1).

Lymphoprep solution (Invitrogen) was used to isolate mononuclear cell fraction and CD34^+^ cells were enriched by using CD34 microbead kit (Miltenyi Biotec). The CD34^+^ and sorted HSC UBC cells were expanded in SFEMII media supplemented with; rhIL-6, rhIL-3 (R&D systems), rhFl3/Flk-2 ligand, rhTPO and rhSCF (R&D systems) all in final concentrations of 20 ng/mL.

### Mice

All mice studies were performed in the pathogen-free animal facility at Karolinska Institutet, Huddinge, Sweden. The animal studies were approved by Linköping Animal Research Ethics Committee, Sweden and the Swedish board of agriculture (Ethical number; ID 530) and all investigations were performed in accordance with national and international guidelines and regulations. The C57BL/6 J wt mice and the NOD-scid IL2Rgnull-3/GM/SF, NSG-SGM3 mice were purchased from The Jackson Laboratory. The Cas9 mice were bought from Jackson Laboratory, stock: Gt(ROSA)26Sortm1.1(CAG-cas9*,-EGFP)Fezh/J.

### Bone marrow transplantation

Congenic non-competitive engraftment was carried out by transplanting 20,000–50,000 cKit^+^ or LSK CD45.1^+^ cells and 200,000 unfractionated CD45.2 supporter cells into lethally radiated (950 cGy) CD45.2^+^ mice via tail vein injection. The level of engraftment of donor cells was investigated at two, five, eight and 16 weeks post transplantation by sampling peripheral blood via the tail vein of transplanted mice. The level of engraftment in the bone marrow was determined by flow cytometric analysis of bone marrow cells extracted from the tibia and femur of euthanised mice eight weeks post transplantation to detect CD45.1 + cells. Mouse xenograft transplantations were performed by sub-lethal radiation (220 cGy) of humanised NSG-SGM3 mice aged six to eight weeks. 50,000–100,000 CD34^+^ UCBs were injected per mouse, six-12 hours post irradiation via tail vein injection. Ciprofloxacin antibiotic was administrated via drinking water to avoid infection after radiation. To determine the level of engraftment in euthanised recipient mice, human UCBs in the BM, tibia and femur were collected at six weeks post transplantation, where engraftment efficiency (hCD45^+^) and cell population frequency were analysed by flow cytometry.

### Lentiviral production and transduction of primary cells

Transfection and transduction were performed as previously described^73^. Briefly, 293-FT cell were transfected with lentiviral targeting, psPAX2 packaging and VSV-G envelope plasmids constructs using the calcium phosphate method. The produced virus particles were harvested 24 h and 48 h after transduction and thereafter concentrated by 16 h centrifugation at 5000 × g. The virus pellet was then washed and re-suspended in media supplemented with growth factors according to the cell culture media requirements of the target cells. Lentiviral transduction was performed by adding the virus supernatant to primary cells maintained in ultra-low attachment plates (Corning) to a final volume of 500 μL. Thereafter, the cells with the virus was spinoculated for two hours at 1000 × g. After centrifugation, the cells were exposed to the virus for 12 hours. After washing, the cells were re-suspended and cultured in fresh SFEMII media supplemented with cytokines for two days. The transduced cells were selected with puromycine (2 ug/mL) for 48 hours.

### Flow cytometry analysis and sorting

To investigate the level of engraftment in BM of transplanted mice, BM cells were harvested from tibia and femur bones and after stained with purified anti-CD16/CD32 (Fc-block), and with anti-mouse lineage markers (CD11b, Gr-1, CD3, CD19 NK1.1 and TER119) together with CD117 (cKit) and Sca-1, for 20 minutes at 4 °C. Cells were washed and resuspended in promidium iodine (PI) (Invitrogen) to visualise live cells. To determine the level of engraftment of human CD45^+^ cells in transplanted NSG-SGM3 mice, BM cells were isolated from tibia and femur. The isolated BM cells were first subjected to Fc-blocking antibodies (ChromPure Mouse IgG, Jackson ImmmunoResearch) and thereafter stained with lineage antibodies (Lin; CD56, CD235a, CD3, CD19, CD11b, CD14) as well as CD34 and CD38. The cells were then incubated with Near-IR Live/Dead marker to detect live cells. Flow cytometric analysis was performed with a 4-laser BD LSRFortessa. To analyse the different mature human cell populations in NSG-SGM3 transplanted mice, the BM cells were isolated and stained with CD11b, CD14, CD56, CD19, CD33, CD16, CD3, HLA-DR and CD45. To exclude dead cells in the analysis, the Live/Dead fixable Aqua dead cells stain kit (Invitrogen) was used.

High resolution sorting of seven fractions of mouse HSCs and progenitor cells was done according to a previously published protocol^56^. For sorting of Lin^−^ cKit^+^ or LSK, BM cells they were first isolated by crushing iliac crest bones, femurae and tibiae of C57BL/6 J mice. cKit^+^ cells were enriched by depletion of mature cells using magnetic Dynabeads (Invitrogen) and purified using antibodies specific for Ter119, B220, Gr1, CD3, Nk1.1 and CD11b. The cells were then stained with flourochrome-conjugated CD117 (cKit), Sca-1 and Lin antibodies. Dead cells were excluded by staining the cells with PI.

To sort HSC UBCs, CD34^+^ cells they were enriched as described above and re-suspended in PBS with 2% FBS and stained with purified anti-human Fc block purified antibody. Thereafter, cells were stained with fluorescence-conjugated antibodies; Lin, CD45, CD34, CD38 antibodies. Dead cells were excluded using PI. Cell sorting was performed on BD FACS Aria III cell sorter or BD FACS FUSION BSL2 using a 85 or 100 microns nozzle. All analysis was performed using FlowJo Version 9.3.3 software (TreeStar). Monoclonal antibodies and conjugations used in this study are listed in Table 1.

**Table 1:** Antibodies used for flow cytometry.

Antibody	Conjugated	Clone	Source
Anti-mouse TER-119	Purified	TER-119	Biolegend
Anti-mouse CD3	Purified	17A2	Biolegend
Anti-mouse/Human CD45R/B220	Purified	RA3-6B2	Biolegend
Anti-mouse Ly-6G/Ly-6C (Gr-1)	Purified	RB6-8C5	Biolegend
Anti-mouse NK1.1	Purified	PK136	Biolegend
Anti-mouse CD11B	Purified	M1/70	Biolegend
Anti-mouse CD16/32	Purified	93	Biolegend
Anti-mouse CD117 (c-kit)	APC-Cy7	2B8	Biolegend
Anti-mouse CD4	PECy5	RM4-5	Biolegend
Anti-mouse CD8a	PECy5	53-6.7	Biolegend
Anti-mouse/Human CD11b (Mac1)	PECy5	M1/70	Biolegend
Anti-mouse CD11c	PECy7	N418	Biolegend
Anti-mouse CD16/32	PECy7	93	Biolegend
Anti-mouse CD19	PE-CF594	1D3	BD
Anti-mouse CD41	FITC	MWReg30	Biolegend
Anti-mouse CD45R/B220	PECy5	RA3-6B2	Biolegend
Anti-mouse CD48	PE	HM48-1	Biolegend
Anti-mouse CD105	Biotin	MJ7/18	Biolegend
Anti-mouse CD117 (cKit)	PerCP-eFluor710	2B8	eBioscience
Anti-mouse CD117 (cKit)	APCeFluor780	2B8	eBioscience
Anti-mouse CD127 (IL7Ra)	Biotin	A7R34	Biolegend
Anti-mouse CD135 (Flt3)	PE	A2F10	Biolegend
Anti-mouse CD150	APC	TC15-12F12.2	Biolegend
Anti-mouse Gr1(Ly6G/Ly6C)	PECy5	RB6-8C5	Biolegend
Anti-mouse Ly-6C	APC	HK1.4	Biolegend
Anti-mouse Ly6D	FITC	49-H4	Biolegend
Anti-mouse NK1.1	PECy5	PK136	Biolegend
Anti-mouse Sca1	PB	D7	Biolegend
Anti-mouse Streptavidin	QD655		Invitrogen
Anti-mouse TER119	PECy5	TER-119	Biolegend
Anti-mouse TER119	PECy5.5	TER-119	eBioscience
Ki-67	FITC	B56	BD
Anti-human CD56	PE-Cy5	HCD56	Biolegend
Anti-human CD235a	PE-Cy5	HIR2	Biolegend
Anti-human CD3	PE-Cy5	HIT3a	Biolegend
Anti-human CD19	PE-Cy5	HIB19	Biolegend
Anti-human CD14	PE-Cy5	6103	Biolegend
Anti-human CD34	APC	581	Biolegend
Anti-human CD38	PE-Cy7	HB7	BD
Anti-human CD45	BV786	HI30	BD
Anti-human HLA-DR	APC-Cy7	L243	Biolegend
Anti-human CD11b	Alexa Fluor488	ICRF44	BD
Anti-human CD14	PerCP-Cy5.5	HCD14	Biolegend
Anti-human CD56	BV605	HCD56	Biolegend
Anti-human CD19	BV786	SJ25c1	BD
Anti-human CD33	PE	WM53	Biolegend
Anti-human CD16	PE-CF594	3G8	BD
Anti-human CD3	PE-Cy5	HIT3a	Biolegend
Anti-human CD45	APC	HI30	Biolegend
Anti NAP1L3	Purified	AA1-506	Antibodies-online
Anti homeobox A3	Purified	AA 414-443	Antibodies-online
Anti homeobox A5	Purified	Polyclonal Middle Region	Antibodies-online
Anti-HOXA9	Purified	AA 245–272	Antibodies-online

### Intracellular protein staining

To determine intracellular protein levels by flow cytometry, we used transcription factor staining buffer set (eBioscience, 00-5523-00). Briefly cells were fixed with fixation buffer for 20 minutes and after washing incubated with permeabilisation buffer for 20 minutes. The cells were incubated with primary antibodies (information about antibodies are listed in Table 1) diluted in permeabilisation buffer, then washed cells stained with secondary antibody (anti-rabbit IgG, F fragment Alexa Flour 647 conjugate, Cell signaling) for 20 minutes. The stained cells were washed and resuspended in PBS + 2% FBS for flow cytometry.

### Cell cycle analysis

UCB HSCs were washed with PBS and stained with first anti-human CD16/32 and then with fluorescence-conjugated anti-human Lin, CD34 and CD38 antibodies. After washing the cells with PBS, they were stained with Live/Dead Fixable Near IR viability dye for 20 minutes at room temperature (RT) and then washed with cold PBS. Cells were fixed with 1% of formaldehyde for 10 min at RT and the crosslinking was stopped with 0.1 M Glycine. 0.05% Triton X-100 in PBS was used to permeabilise the cells and after washing the cells once in PBS, they were incubated in 10 uL of Ki67 for 2 hours at 4 °C in the dark. Washed cells were resuspended in 0.5 μg/mL DAPI for 20 min at RT before FACS analysis using Fortessa LSRII (BD).

### Serial replating assays

Transduced cells were selected with Puromycine in concentration of 2 μg/ml for 48 hours and thereafter resuspended in Methocult H4435 (Stemcell technology) at concentration of 1 cell in 150 μl per well in 96-well plates. After 14 days, the colonies from each well were washed with PBS, resuspended in 20 μl of IMDM and cultured in new 96-well plate contain 150 μl Methocult.

### Apoptosis analysis

After staining THP-1 cells with fluorescence-conjugated antibodies and Live/Dead fixable marker as described above, the cells were washed and re-suspended in Annexin-binding buffer (BioLegend) containing Annexin V FITC (BioLegend) for 20 minutes AT RT. After another round of washing the cells were re-suspended in Annexin-binding buffer and analysed by flow cytometric analysis. The absolute number of human cells was analysed by a high-throughput automated plate reader (BD LSRFortessa).

### Vectors and Molecular Cloning

The inducible gRNA vectors were generated as previously described^74^. Briefly, gRNAs were designed using Optimised CRISPR Design - MIT (http://crispr.mit.edu), and subsequently cloned into the inducible vector pRSITEP-U6Tet-(sh)-EF1-TetRep-2A-Puro (Cellecta). PCR amplification using primers homologous to upstream and downstream genomic regions of the *NAP1L3* gRNA target site were used to generate DNA fragments that were sub-cloned for sequencing of in total 10 individual clones. For cloning *Nap1l3* cDNA into an overexpression vector, cDNA was purchase from Thermoscientific and PCR amplified by primers providing overhangs with restriction enzyme sites; XbaI, NotI. After cutting, the cDNA was cloned into pCHD-the MCS-EF1 lentivector from Biocat (pCDH-MSCV-MCS-EF1-GFP-Puro, Cat. No. CD713B-1). All oligos used in the study are listed in Table 2.

**Table 2:** Oligos used in the studies.

Target site	Application	Forward oligo (5′ > 3′)	Reverse oligo (5′ > 3′)
mNap1l3*	qPCR		
mHprt1**	qPCR		
h*NAP1L3*	qPCR	ACCAGAGGTGAAAGCTGAA	CCTGGGGGACTTCTTTAGGA
h*UBC*	qPCR	CTGGAAGATGGTCGTACCCTG	GGTCTTGCCAGTGAGTGTCT
h*HOXA3*	qPCR	CTCCAGCTCAGGCGAAAG	ACAGGTAGCGGTTGAAGTGG
h*HOXA4*	qPCR	CATGTCAGCGCCGTTAACC	ACCTGCTGCCGGGTGTAG
h*HOXA5*	qPCR	CGCCCAACCCCAGATCTAC	CCGCCTATGTTGTCATGACTTATG
h*HOXA6*	qPCR	CGCGGGTGCTGTGTATG	CCTTCTCCAGCTCCAGTGTC
h*HOXA9*	qPCR	CCCCATCGATCCCAATAACC	CCAGGGTCTGGTGTTTTGTATAGG
h*CDK1*	qPCR	GGAAACCAGGAAGCCTAGCA	GATCATAGATTAACATTTTCGAGAGCAA
*mNAP1l3*	OE cloning	ANGCTCTAGAATGGCAGAAGCGGATCCTAAAATG	ANGCGCGGCCGCCTACTTGTAGTACTTCCTATTT CATAAT
*mNAP1l3*	CRISPR gRNA	ACCGGCGCCTCTTCAGCAACCCCAT	AAACATGGGGTTGCTGAAGAGGCGC
*mNAP1l3*	PCR mutation analysis	CCACAGCTGCTGCAGTCC	GCCCTTCTGGAAGGCTCAG
*mNAP1l3*	Sequencing mutation analysis	CAGTCCGTGTTGCCACTG	

*m*Nap1l3* Taqman probes was purchased from ThermoFisher Scientific (Mm01214875_s1).

**m*Hrpth* Taqman probes was purchased from ThermoFisher Scientific (Mm03024075_m1).

### CFU-C assay

The sorted normal mouse (cKit^+^/LSK) and human BM (Lin^−^CD34^+^CD38^−^) were seeded in the MethoCult semi-solid media (Stem Cell Technologies) for mouse 150–300 cells/1 cm^2^ dish (M3434) and for human (M4435) 200–400 cells/1 cm^2^ dish for 10–12 days (mouse) or 12–14 days (human) respectively. The colonies were stained with Giemsa (Sigma) and scored under microscope, where a cluster of more than 50 cells was defined as one colony.

### RNA-Sequencing

Total RNA was extracted using RNeasy Micro Kit (Qiagen) from 20 × 10^5^ FACS-sorted Lin^−^CD34^+^CD38^−^ UCBs. TotalScript^TM^ RNA-seq kit (Epicentre, Madison, WI) was used to create strand specific pair-end RNA libraries according to the manufacturer’s instructions. Libraries were sequenced by using the Illumina platform. RNA-Seq reads were mapped to the Ensembl Homo_sapiens and GRCh38 reference genome using the STAR aligner. Gene assignment was performed using featureCounts. Normalisation and the sample group comparisons were performed using DESeq. 2. GSEA analysis was performed to analyse the enrichment of the gene sets following the developer’s protocol 60 (http://www.broad.mit.edu/gsea/). The Biocarta gene sets (CP) were used to identify pathways significantly associated with genes that were up or downregulated as a result of *NAP1L3* downregulation in UCBs. As an approach to identify correlations to defined biological states or processes the Hallmark gene sets (H) was used to analyse enrichments of gene sets.

### Statistical analysis

Τhe unpaired t-tests for the statistical analysis of our data were performed using GraphPad Prism 6.

### Data availability

The datasets generated during and/or analysed during the current study are available in the GEO repository: https://www.ncbi.nlm.nih.gov/geo/query/acc.cgi?acc=GSE106170.

## Electronic supplementary material

Supplementary Information

Supplementary Table 1
