# Supplementary material for: The histone chaperone NAP1L3 is required for haematopoietic stem cell maintenance and differentiation
Source: Sci Rep. 2018 Jul 25;8:11202. doi: 10.1038/s41598-018-29518-z (PMC6060140; doi:10.1038/s41598-018-29518-z)
Supplement: Supplementary file 1 — Supplementary Information [file 41598_2018_29518_MOESM1_ESM.pdf]

Supplementary Information

Article in *Scientific Reports*

The histone chaperone NAP1L3 is required for haematopoietic stem cell maintenance and differentiation

Yaser Heshmati, Shabnam Kharazi, Gözde Türköz, David Chang, Esmat Kamali Dolatabadi, Johan Boström, Aleksandra Krstic, Theodora Boukoura, Emma Wagner, Nadir Kadri, Robert Månsson, Mikael Altun, Hong Qian, Julian Walfridsson.

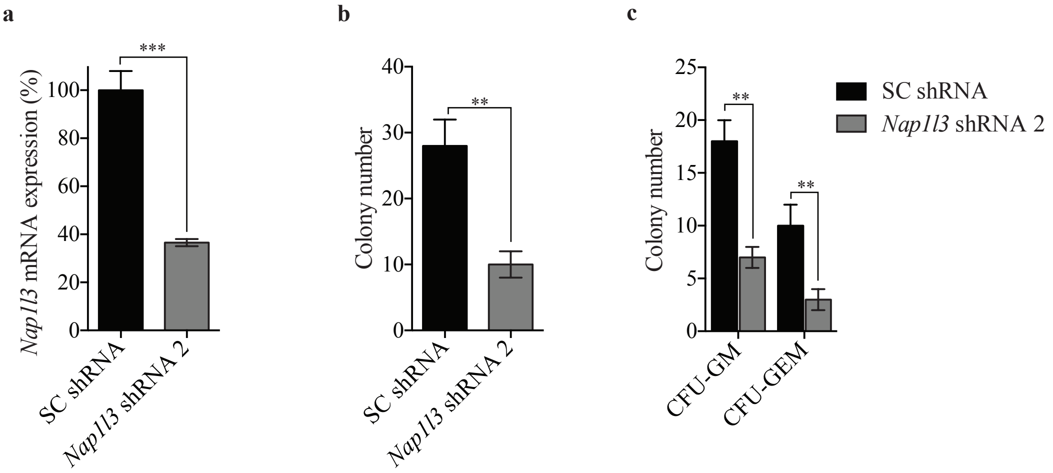

Supplementary Figure 1. Nap1l3 downregulation impairs colony-forming capacity.
A. qPCR analysis showing Nap1l3 mRNA levels (normalized to Hprt) of sorted LSK HSCs transduced with an shRNA against Nap1l3 (Nap1l3 shRNA), or a control vector (SC shRNA). The data is represented as the mean ±s.e.m, **p<0.01, ***p<0.005 (unpaired t-test), n=3. **B** and **C.** Bar graph showing the total colony numbers (B), and colony numbers of CFU-GM and CFU-GEM (C), formed from LSK HSCs transduced with Nap1l3 shRNA (Nap1l3 shRNA) or a control vector (SC shRNA) after ten days of clonal growth in methylcellulose. **p<0.01, ***p<0.005, ****p<0.001 (unpaired t-test), n=3.

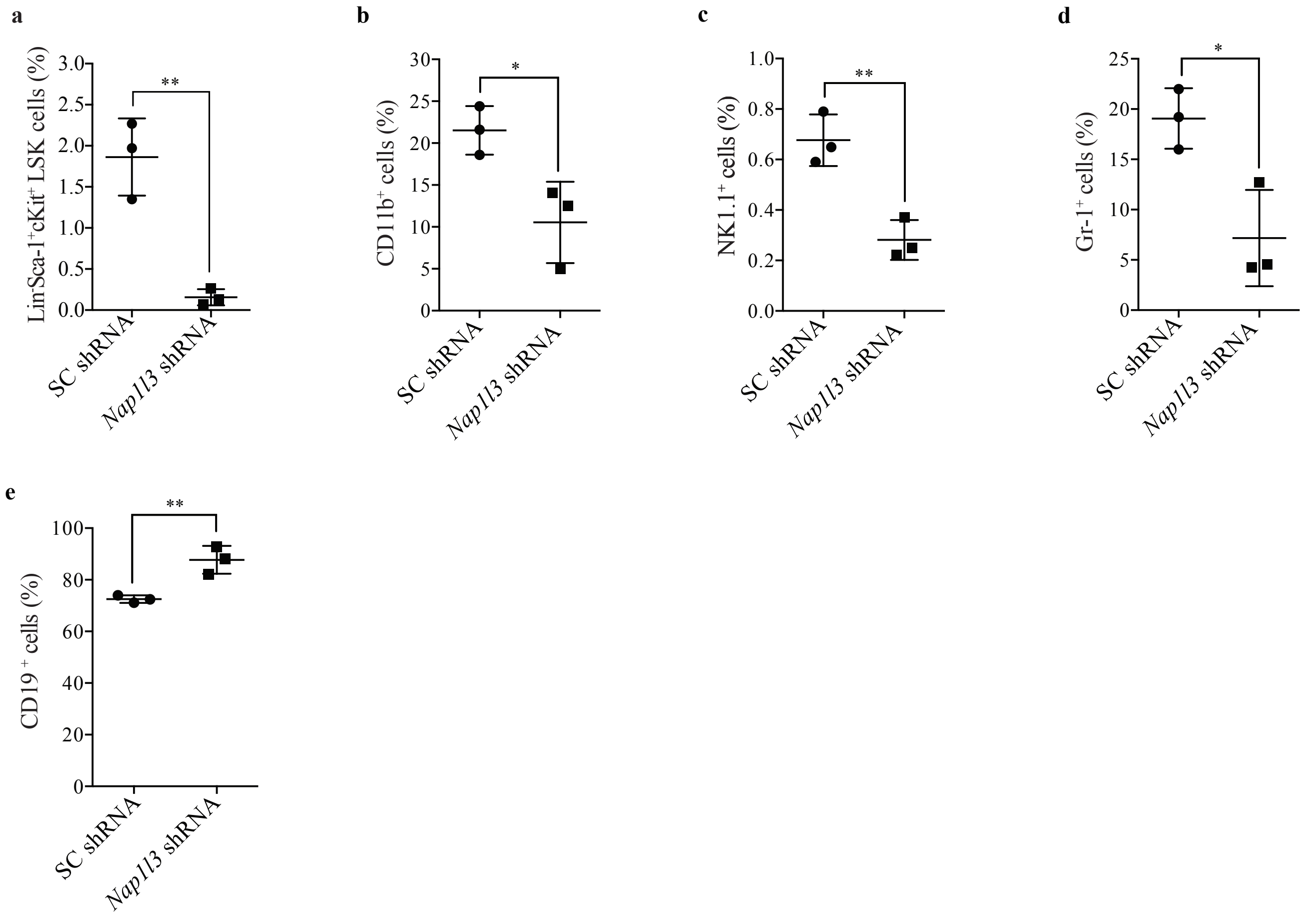

Supplementary Figure 2. Nap1l3 downregulation impairs maintenance of murine HSCs and blood lineage regeneration.

Percentage of donor derived LSK HSCs (A), or mature donor cells; CD11b+ myeloid (B), NK1.1+ NK cells (C), Gr-1+ granulocytes (D), and CD19+ B cells (E), transduced with shRNAs against Nap1l3 or a control vector isolated from the bone marrow of recipient mice eight weeks after transplantation. The bone marrow cells were analysed by flow cytometry. *p<0.05, **p<0.01 (unpaired t-test), n=3.

a b c

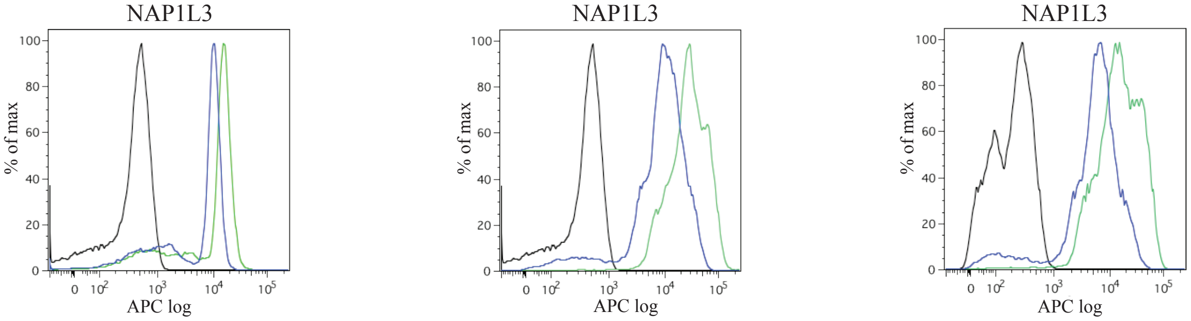

a

	Sample	MFI
■	SC shRNA	12487
■	*NAP1L3* shRNA	7707

b

	Sample	MFI
■	SC shRNA	36972
■	*NAP1L3* shRNA	12952

c

	Sample	MFI
■	SC shRNA	22036
■	*NAP1L3* shRNA	8057

Supplementary Figure 3. shRNA-mediated downregulation results in significant reduction in protein levels of NAP1L3.

Three independent flow cytometry plots showing quantification of intracellular NAP1L3 protein levels from sorted (Lin-CD34+CD38-) UCB HSCs transduced with an shRNA vector against NAP1L3 (NAP1L3 shRNA) or a control vector (SC shRNA). The mean of the MFI of the three independent experiments are shown in Figure 3B.

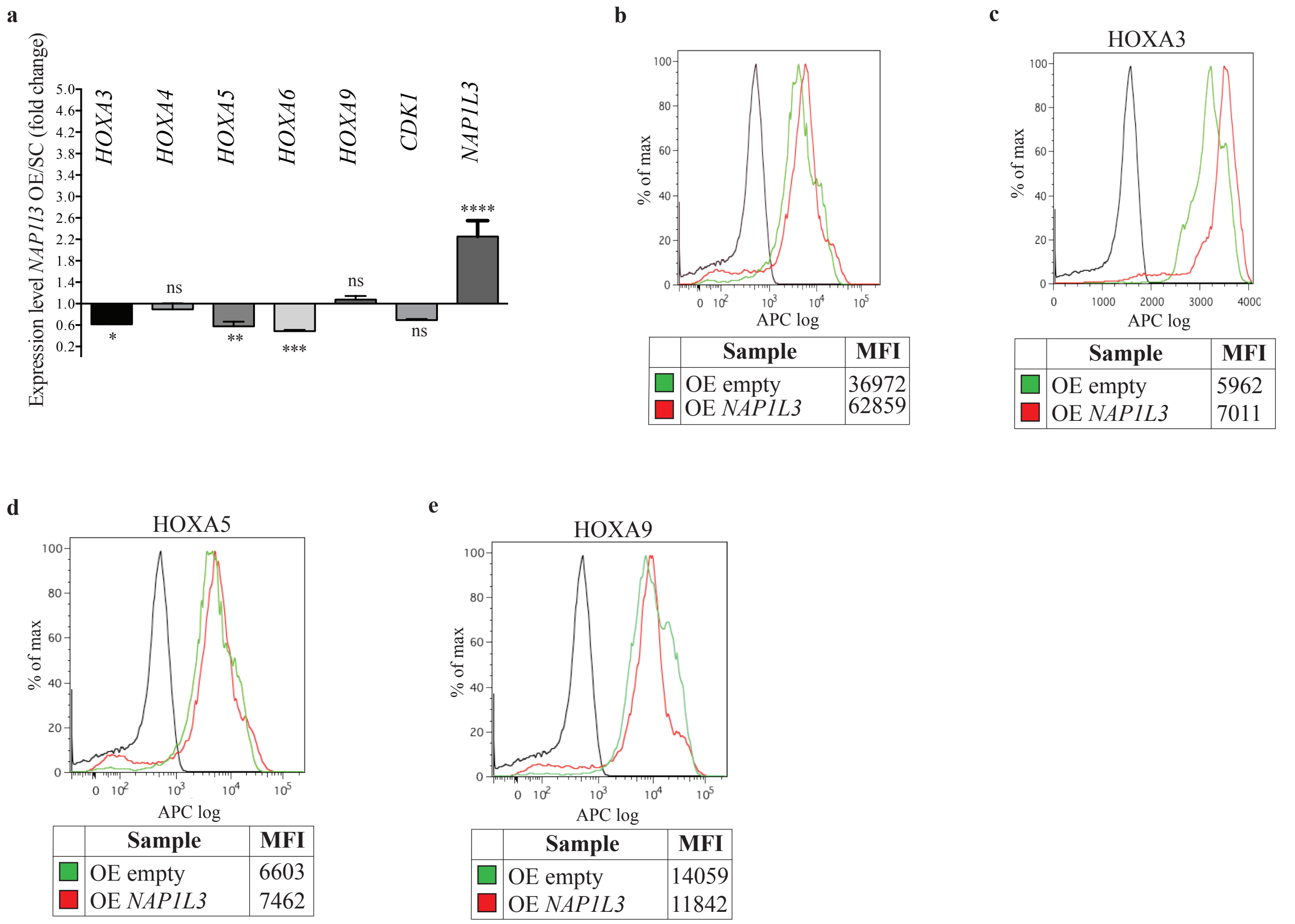

Supplementary Figure 4. Enforced expression of NAP1L3 do not affect expression of HOXA genes. **A.** qPCR analysis of mRNA levels of genes showing changes in expression in RNA-Seq data of CD34+ HSPC UCBs cells transduced with a lentiviral vector expressing NAP1L3 (OE NAP11L3), relative to control cells transduced with an empty vector (OE empty vector), 72 hours post transduction. The data is represented as the mean ±s.e.m., ns= non significant, *p<0.05 , **p<0.01 , ***p<0.0015 (unpaired t-test), n=3. **B-E.** Flow cytometric quantification of mean fluorescence intensity (MFI) of intracellular NAP1L3 (B), HOXA3 (C), HOXA5 (D) and HOXA9 (E), protein levels, of CD34+ HSPC UCBs cells transduced with shRNA against NAP1L3 or cells transduced with control vectors, 72 hours post transduction.
